# Anti-Inflammatory, Antinociceptive, and Gastric Effects of *Hypericum perforatum* in Rats

**DOI:** 10.1100/tsw.2005.78

**Published:** 2005-08-08

**Authors:** Omar M.E. Abdel-Salam

**Affiliations:** Department of Pharmacology, National Research Center, Tahrir St., Dokki, Cairo, Egypt

**Keywords:** Hypericum perforatum, inflammation, nociception, gastric ulcer

## Abstract

The pharmacological activity of *Hypericum perforatum* was assessed using models of inflammation, nociception, and gastric mucosal injury in rats. *H. perforatum* was given systemically as well as orally. When administered systemically, *H. perforatum* (50–300 mg/kg, s.c.) produced a dose-related and significant inhibition of the edematogenic response to s.p. injection of carrageenan. The percentages of maximal inhibition by the above doses were 53.7, 61.3, and 75.3%, respectively (compared to 90% after 50 mg/kg fluoxetine and 60.7% after 72 mg/kg etodolac). In tests of nociception, *H. perforatum*, administered orally, displayed antinociceptive activity in the tail electric stimulation and hot plate tests. The antinociceptive activity was observed with 25 mg/kg and a maximal increase in hot plate latency by 50% (compared to 73.2 and 77.8% increases by 5 or 10 mg/kg fluoxetine, respectively). In contrast, the acetic acid–induced (0.6%, i.p.) writhing was significantly reduced by fluoxetine or etodolac, but not *H. perforatum*. Also, the nociceptive response caused by i.p. injection of capsaicin (1.6 μg/paw) was unaffected by *H. perforatum*, but reduced by fluoxetine. Injection of *H. perforatum* (50, 125, or 250 mg/kg, s.c.) to pylorus-ligated rats, decreased gastric acid secretion, but increased indomethacin-induced gastric mucosal lesions dose dependently. These results demonstrate that H. perforatum exhibits antiedematogenic and antinociceptive properties, which may be of value for the management of inflammatory painful conditions. The agent, however, causes gastric irritation and may aggravate that of NSAIDs.

## INTRODUCTION

Extracts of *Hypericum perforatum* (St. John's wort) have gained much interest for their antidepressant effects. Studies have suggested that *H. perforatum* may be of comparable efficacy to the selective serotonin reuptake inhibitor (SSRI) sertraline or to the tricyclic antidepressant imipramine[1,2]. Tricyclic antidepressants are the mainstay in the management of neuropathic pain syndromes[3,4]. These agents achieve a good or moderate response, but their therapeutic utility has been limited by adverse events[5,6]. Studies have suggested anti-inflammatory and analgesic properties for *H. perforatum*[7,8]. *In vitro*, iNOS

and COX-2 expression, as well as myeloperoxidase activity and the generation of reactive oxygen species, were inhibited by the agent[8,9,10].

The identification of compounds that would alleviate neuropathic pain still represents a challenging goal. It was the aim of the present study to assess the effects of *H. perforatum* extract in animal models of pain and acute inflammation. The effects of *H. perforatum* were compared with those of fluoxetine, a potent SSRI, and the selective COX-2 inhibitor etodolac. These agents displayed marked anti-inflammatory and analgesic properties[11,12,13]. In addition, the gastric effects of *H. perforatum* in the presence of indomethacin, a nonsteroidal anti-inflammatory drug (NSAID) commonly used in many arthritic and inflammatory conditions, were evaluated.

## MATERIALS AND METHODS

### Animals

Sprague-Dawley strain rats weighing 120–130 g of body weight (National Research Centre, Cairo) were used. Unless otherwise indicated, food and water were provided *ad libitum*. All animal procedures were performed in accordance to the Institutional Ethics Committee and in accordance with the recommendations for the proper care and use of laboratory animals (NIH publication No. 85–23, revised 1985).

### Drugs

A commercially available St. John's wort (*H. perforatum*) extract (Safamood, ATOS Pharma, ARE) was used and dissolved daily in 5 ml of distilled water and 1.76 ml of alkaline solution (NaOH; pH = 13)[10]. The stock solution was further diluted before use to achieve the doses needed. Fluoxetine hydrochloride (Amoun Pharmaceutical Co., Cairo, ARE), indomethacin (Kahira Pharm & Chem. IND Co., Cairo, ARE), etodolac (Napilac, Global Napi Pharmaceuticals, ARE), carrageenan, and capsaicin (Sigma, USA) were used. Stock solutions of capsaicin (10 mg/ml) contained 10% ethanol, 10% Tween 80, and 80% saline solution. Analytical-grade glacial acetic acid (Sigma) was diluted with pyrogen-free saline to provide a 0.6% solution for i.p. injection. *H. perforatum* was dissolved in distilled water.

### Tests of Inflammation

#### *Carageenin-Induced Paw Edema*

Paw swelling was elicited by s.p. injection of 100 µl of 1% sterile lambda carrageenan suspension in saline into the right hind paw[14]. Contralateral paw received an equal volume of saline. The edema component of inflammation was quantified by measuring the increase in paw volume (ml) with a plethysmometer (Ugo Basile, Milan, Italy) before carrageenan injection and at selected times thereafter. Edema was expressed as a percentage of change from control (predrug) values.

The effect of an acute administration of *H. perforatum* (50, 150, or 300 mg/kg, s.c., 0.2 ml/rat, n = 6/group) was studied and compared with that of the SSRI fluoxetine (fluoxetine at 50 mg/kg, s.c.) or the COX-2 inhibitor etodolac (18, 36, or 72 mg/kg, s.c., 0.2 ml/rat, n = 6/group). Drugs were administered 30 min before the injection of the carrageenan suspension. The control groups received saline (0.2 ml/rat, n = 6/group; s.c.) instead.

## Tests of Nociception

### Hot Plate Assay

The hot plate test was performed using an electronically controlled hot plate (Ugo Basile, Italy) heated to 52°C (±0.1°C). The cutoff time was 30 s. The latency until rats showed first signs of discomfort (hind paw lifting, hind paw licking, or jumping) was recorded, before (baseline) and at 1 or 2 h after the administration of *H. perforatum* (25, 50, or 100 mg/kg, p.o., 0.2 ml/rat, n = 6–7/group), fluoxetine (5 or 10 mg/kg, s.c., n = 6/group), or etodolac (18, 36, or 72 mg/kg, s.c., n = 6/group). The experimenter was blind to treatment and dose.

### Tail Electric Stimulation Test

Groups of rats (n = 6/group) were given *H. perforatum* (50, 150, or 300 mg/kg, p.o.) or saline (control). Other groups were treated with fluoxetine (5 or 10 mg/kg, p.o., n = 6/group) or etodolac (18, 36, or 72 mg/kg, p.o., n = 6/group). The minimum current required to elicit vocalization on electrical stimulation of the tail was determined for the control and drug-treated groups[15]. Electrical stimulation of the tail was applied by means of 515 Master Shocker (Lafayette Inst. Co.). Stimulation was carried out by an alternative current of 50 cycle/s for 0.2 s.

### Capsaicin-Induced Hind Paw Licking

*H. perforatum* (25 or 50 mg/kg), fluoxetine (5 or 10 mg/kg), etodolac (18 or 36 mg/kg), or saline was given p.o., 1 h before injection of capsaicin (1.6 μg/paw; 25 μl) under the skin of the dorsal surface of the right hind paw. Observation started after capsaicin injection and lasted for 5 min. The time the animals spent licking the injected paw was determined using a stopwatch[16].

### Acetic Acid–Induced Writhing

*H. perforatum* (25 or 50 mg/kg), fluoxetine (5 or 10 mg/kg), etodolac (18 or 36 mg/kg), or saline was given p.o., 1 h before i.p. injection of 0.6% acetic acid (0.4 ml)[17]. The number of writhes (constriction of abodomen, twising of trunk, and extension of hind legs) during 20-min observation period was noted.

## Gastric Ulcerogenic Studies

Rats were fasted for 18 h with free access to water. Pylorus ligation was done under light ether anesthesia, then gastric mucosal damage was evoked by indomethacin (20 mg/kg, s.c.). Rats received either saline (0.2 ml/rat, s.c., n = 6) (control) or *H. perforatum* (50, 125, or 250 mg/kg, 0.2 ml/rat, s.c., n = 6–7/group) and 2 ml of saline into their stomachs. Rats were killed 4 h later. Gastric acid output determined by titration to pH 7.0 with 0.01*N* NaOH and $H^+$ output expressed as μEq/4 h. Gastric mucosal lesions were scaled as described earlier[18]. In addition, the effect of *H. perforatum* (200 mg/kg, 0.2 ml/rat, s.c., n = 6) on gastric acid secretion was examined in pylorus-ligated rats.

## Statistical Analyses

Data are expressed as mean ± S.E. Differences between vehicle control and treatment groups were tested using one- and two-way ANOVA followed by multiple comparison by the Duncan's multiple comparison test. When there were only two groups a two-tailed Student's *t* test was used. A two-tailed probability value less than 0.05 was considered statistically significant.

## RESULTS

### Anti-Inflammatory Effects of *H. perforatum*

*H. perforatum* dose dependently inhibited the carrageenan-induced inflammatory edema (two-way ANOVA: treatment effect: $F_{3,\ 84} = 175.6$; $p < 0.0001$; time effect: $F_{3,\ 84} = 91.9$; $p < 0.0001$). The antiedema effect was maximal 1 h after carrageenan injection, with 53.7, 61.3, and 75.3% inhibition of edema formation by 50, 100, or 300 mg/kg of the extract, respectively. Fluoxetine (50 mg/kg) caused more pronounced inhibition of paw edema (90.5, 88.6, 85.4, and 84% at 1, 2, 3, and 4 h postcarrageenan, respectively) (Fig. 1A). Fluoxetine caused significanly less edema than *H. perforatum* at all time points. Significant inhibition of edema was also obtained with etodolac (two-way ANOVA: treatment effect: $F_{3,\,80} = 66.2$; $p < 0.001$; time effect: $F_{3,\,80} = 67.1$; $p < 0.001$; drug × time interaction: $F_{9,\,80} = 8.2$; $p < 0.01$). Edema was reduced by 22.8, 19.1, and 24.7% (18 mg/kg etodolac); by 33.8, 42, and 42.5% (36 mg/kg etodolac); or 43.3, 51.9, and 60.7% (72 mg/kg etodolac); at 2, 3, and 4 h postcarrageenan, respectively (Fig. 1B).

Fig. 1A

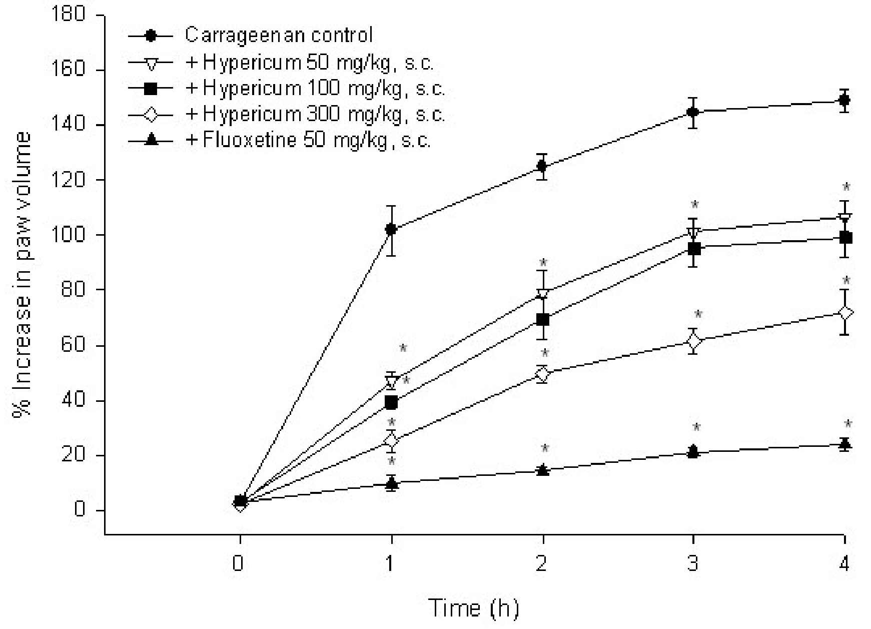

A

**FIGURE 1.** The antiedema effect of *H. perforatum* extract, fluoxetine (A) and etodolac (B). Results are expressed as a percentage change from control (predrug) values, each point represents mean ± S.E of 6 rats per group. Asterisks indicate significant change from control value at respective time points (ANOVA and Duncan's multiple comparison test).

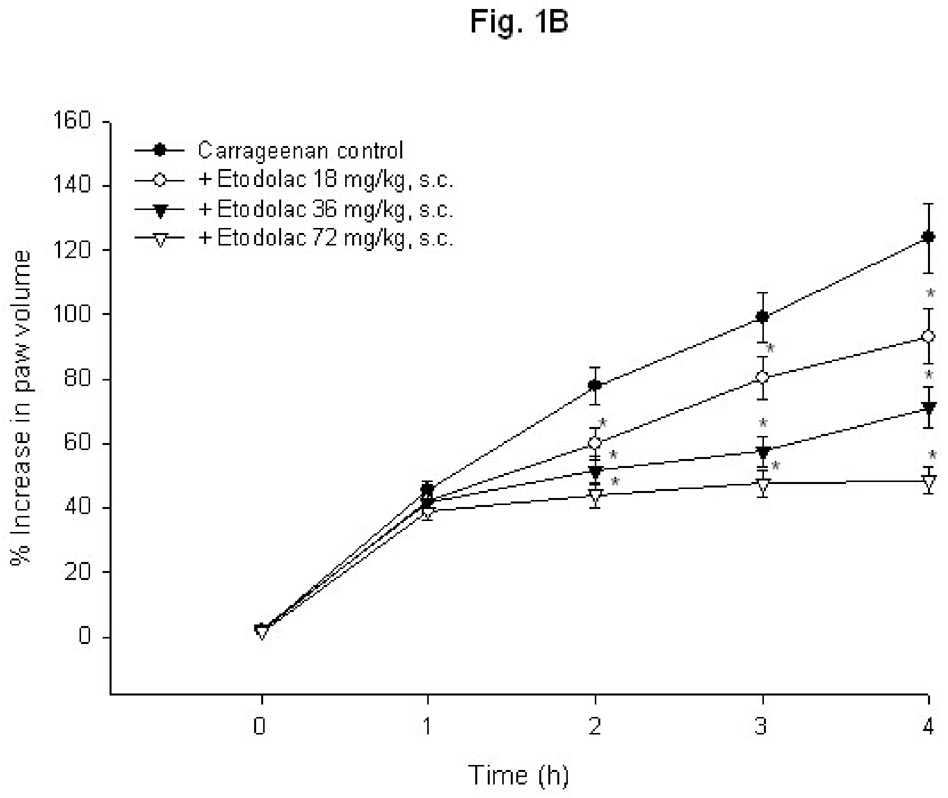

**FIGURE 1B**

## Antinociceptive Effects of *H. perforatum*

### *Hot Plate Assay*

The reaction time on the hot plate was delayed by *H. perforatum*. The antinociceptive effect of the agent was produced with a 25 mg/kg and maximal increase in hot plate latency by 50% 2 h after injection. In comparison, 73.2 and 77.8% increases in hot plate latency were observed after 5 or 10 mg/kg fluoxetine, respectively. Meanwhile, an increase in hot plate latency by about 38.8% was seen after treatment with etodolac at 36 or 72 mg/kg (Table 1).

### *Tail Electric Stimulation Test*

*H. perforatum* (50, 150, or 300 mg/kg) produced a significant rise in electrical current threshold in the tail stimulation test in rat by 16.1, 24.9, and 27.1% and 19.9, 36.9, and 37.7% vs. control values, 1 and 2 h postdrug, respectively. A significant rise in nociceptive thresholds by 31.3 and 42.5% was obtained with fluoxetine 2 h following administration. Etodolac at the highest dose examined of 72 mg/kg significanly increased the nociceptive threshold by 21.8% for 1 h postinjection (Table 2).

### *Capsaicin-Induced Hind Paw Licking*

The duration of paw licking following i.p. capsaicin injection was unaffected by prior administration of *H. perforatum* or etodolac, but reduced by 18.9 and 20.4% after 5 or 10 mg/kg fluoxetine, respectively (Table 3)

.

**TABLE 1**
**Antinociceptive Activity of *H. perforatum*, Fluoxetine, and Etodolac in the Hot Plate Test**

| | 0 Time (basal) | 1 h | 2 h | 1 h | 2 h |
|---|---|---|---|---|---|
| | | | | % Change | |
| *H. perforatum* | | | | | |
| 25 mg/kg | 12.62 ± 0.89 | 16.42 ± 0.64** | 18.95 ± 1.7** | 30.1 | 50.2 |
| 50 mg/kg | 12.60 ± 1.14 | 17.45 ± 1.32* | 18.15 ± 1.3** | 38.5 | 44.0 |
| 100 mg/kg | 12.82 ± 1.20 | 18.75 ± 1.86* | 18.68 ± 1.1** | 46.3 | 45.7 |
| Fluoxetine | | | | | |
| 5 mg/kg | 11.55 ± 1.4 | 16.13 ± 1.22* | 20.0 ± 2.1** | 39.7 | 73.2 |
| 10 mg/kg | 11.25 ± 1.6 | 19.80 ± 2.1** | 20.0 ± 1.7** | 76.0 | 77.8 |
| Etodolac | | | | | |
| 18 mg/kg | 11.30 ± 0.78 | 13.46 ± 1.1 | 13.61 ± 0.98 | 19.1 | 20.4 |
| 36 mg/kg | 10.80 ± 0.91 | 13.60 ± 1.5 | 15.0 ± 1.2* | 25.9 | 38.9 |
| 72 mg/kg | 11.82 ± 0.8 | 15.22 ± 1.1* | 16.4 ± 1.1** | 28.8 | 38.8 |

Shown are basal and drug-induced (1- and 2-h measurements) latencies for the nociceptive reaction. Data are expressed as means and S.E.M. (n = 6/group). Asterisks indicate significant increase in nociceptive latencies (*: $p < 0.05$; **: $p < 0.01$) compared with the basal level of nociceptive reaction (Student's *t* test).

**TABLE 2**
**Antinociceptive Activity of *H. perforatum* in the Tail Electric Stimulation Test in Rat**

| | 1 h | 2 h | 1 h | 2 h |
|---|---|---|---|---|
| | | | % Change | |
| Control | 283.7 ± 20.5 | 261.4 ± 13.6 | | |
| *H. perforatum* | | | | |
| 50 mg/kg | 329.3 ± 11.7* | 313.6 ± 19.8* | 16.1 | 20.0 |
| 150 mg/kg | 357.9 ± 8.9* | 354.3 ± 15.4* | 26.2 | 35.5 |
| 300 mg/kg | 360.7 ± 12.8* | 360.0 ± 13.6* | 27.1 | 37.7 |
| Control | 292.0 ± 19.7 | 288.5 ± 19.2 | | |
| Fluoxetine | | | | |
| 5 mg/kg | 339.8 ± 16.1 | 378.8 ± 16.5* | 16.4 | 31.3 |
| 10 mg/kg | 352.2 ± 18.0* | 411.0 ± 19.5* | 20.6 | 42.5 |
| Control | 302 ± 18.7 | 311.0 ± 20.3 | | |
| Etodolac | | | | |
| 18 mg/kg | 337.0 ± 19.6 | 322.0 ± 21.0 | 11.6 | 3.5 |
| 36 mg/kg | 343.2 ± 17.9 | 344 ± 18.3 | 13.6 | 10.6 |
| 72 mg/kg | 368.0 ± 19.0* | 348.0 ± 23.5 | 21.8 | 11.9 |

Shown are control and drug-induced (1- and 2-h measurements) changes for the nociceptive reaction. Data are expressed as means and S.E.M. (n = 6/group). Asterisks indicate significant rise in electrical current threshold (microA) ($p < 0.05$) compared with the control level of nociceptive reaction (one-way ANOVA, Duncan test).

**TABLE 3**
**Effect of *H. perforatum*, Fluoxetine, and Etodolac on Duration of Capsaicin-Induced Paw Licking and Number of Writhes in the Acetic Acid Test in Rat**

| Drugs | Capsaicin Test | Acetic Acid Test |
|---|---|---|
| Control | 98.3 ± 7.7 | 13.83 ± 1.27 |
| *H. perforatum* | | |
| 25 mg/kg | 92.7 ± 6.1 | 16.2 ± 1.0 |
| 50 mg/kg | 112.7 ± 6.5 | 14.83 ± 1.2 |
| Control | 84.5 ± 5.8 | 17.5 ± 0.92 |
| Fluoxetine | | |
| 5 mg/kg | 68.4 ± 5.1* | 7.0 ± 0.52* |
| 10 mg/kg | 67.3 ± 4.4* | 5.0 ± 0.68* |
| Control | 82.7 ± 6.1 | 15.7 ± 1.1 |
| Etodolac | | |
| 18 mg/kg | 75.2 ± 5.8 | 12.2 ± 0.87* |
| 36 mg/kg | 70.5 ± 4.6 | 8.5 ± 0.76* |

Data are expressed as means and S.E.M. (n = 6/group). Asterisks indicate significant decrease in nociceptive reaction ($p < 0.05$) compared with the control group. Rats treated with 36 mg/kg etodolac showed significantly less writhes in the acetic acid compared with those given 16 mg/kg (one-way ANOVA, Duncan test).

### *Acetic Acid-Induced Writhing Test*

Writhing was unaffected by *H. perforatum,* but significantly reduced by fluoxetine or etodolac (Table 3).

## Gastric Effects of *H. perforatum*

### *Gastric Acid Secretion*

Gastric acid secretion decreased by 17% in rats given *H. perforatum* ($p < 0.05$, Student's $t$ test). The volume of gastric secretion was also significantly reduced by 32.6% by the drug (3.1 ± 0.36 vs. 4.6 ± 0.18 ml/rat, $p < 0.05$, Student's $t$ test ).

### *Indomethacin-Induced Gastric Lesions*

*H. perforatum* increased the number and severity of gastric mucosal lesions evoked by indomethacin, in a dose-dependent manner. Gastric acid secretion decreased by 17.7, 29.2, and 53.1% in rats treated with *H. perforatum* (Table 4).

**TABLE 4**
**Effect of *H. perforatum* Extract on Gastric Mucosal Injury Induced by Indomethacin in Pylorus-Ligated Rats**

| Treatment Group | Number of Lesions | Severity of Lesions | Gastric Secretory Volume (ml/4 h) | Gastric Acid Output (μEq/4 h) |
|---|---|---|---|---|
| IND control | 2.0 ± 0.52 | 4.0 ± 0.86 | 7.67 ± 0.56 | 392.67 ± 24.84 |
| + *H. perforatum* | | | | |
| 50 mg/kg | 5.34 ± 1.12 | 6.67 ± 1.05 | 7.18 ± 0.39 | 323.16 ± 29.9* |
| 125 mg/ kg | 7.34 ± 1.22* | 9.0 ± 0.82* | 7.08 ± 0.35 | 278.17 ± 16.5* |
| 250 mg/kg | 9.42 ± 1.92* | 15.71 ± 2.0* | 4.37 ± 0.61* | 184.0 ± 16.98* |

Data are expressed as means and S.E.M. (n = 6–7/group). Gastric mucosal damage was evoked in pylorus-ligated rats by s.c. administration of 20 mg/kg of indomethacin (IND). *H. perforatum* dissolved in saline was s.c. administered (0.2 ml/rat). Rats were killed 4 h after pylorus ligation and drug administration. Statistical comparison of the difference between the control group and treated groups is indicated by asterisks; * = $p < 0.05$ (one-way ANOVA and Duncan's multiple range test). Gastric acid output was significantly lower in rats treated with all doses of *H. perforatum* compared with the control group, with those given the drug at 250 mg/kg having significantly lower $H^+$ output and gastric secretory volume than all other groups. The severity of gastric mucosal lesions was significantly higher in rats given 250 mg/kg of *H. perforatum* compared with all other groups in the study.

## DISCUSSION

The present study confirms and extends other work that *H. perforatum* extract exerts both anti-inflammatory and antinociceptive effects. In this respect, the agent inhibited paw edema caused by the injection of carrageenan in rat. The effect was dose dependent, with a maximal reduction in edema of 75.3%, a value that is even higher than that obtained with the COX-2 inhibitor etodolac in maximal doses. The antiedema effect of *H. perforatum* was marked in the first hour following drug administration. This could suggest interference with the actions of histamine, serotonin, or kinins, the inflammatory mediators implicated in the early phase of the carrageenan-induced inflammatory response[19]. *H. perforatum* showed, in addition, marked antinociceptive properties when examined in the hot plate assay and tail electric stimulation test. These effects were also exhibited by fluoxetine and etodolac. The former, an SSRI, has been shown to possess marked anti-inflammatory and analgesic properties[11,12,13]. In a model of neuropathic pain in rat, etodolac, a COX-2 inhibitor, alleviated heat-evoked hyperalgesia[12].

*H. perforatum* was ineffective, however, in the acetic acid–induced writhing response, which was significantly reduced by fluoxetine or etodolac. The writhing response to acetic acid is brought about by the release of prostacyclin synthesized by cyclo-oxygenase in the abdominal cavity of the mice[20]. It is reduced by cyclo-oxygenase inhibitors such as meloxicam or diclofenac[21], by morphine[22], and also by fluoxetine[13]. *H. perforatum,* also and in contrast to fluoxetine, did not have antinociceptive properties against neurogenic pain induced by C-fiber excitation with i.p. capsaicin.

Antidepressants increase noradrenaline and serotonin concentrations at the synapse, an action thought to involved their pain-alleviating properties[23]. The antinociceptive action of systemically administered antidepressants can be inhibited by the opioid receptor antagonist naloxone, suggesting the involvement of opioid sensitive pathways[24]. Extracts of *H. perforatum,* on the other hand, do not fit into the action of classic tricyclic antidepressants or the SSRIs. The drug acts to inhibit the reuptake of several neurotransmitters, including serotonin, norepinephrine, dopamine, gamma-aminobutyric acid, and L-glutamate[25,26].

In the present study, administration of *H. perforatum* inhibited gastric acid secretion in pyloric-ligated rats, yet exacerbated gastric lesions caused by indomethacin. A local irritant action is unlikely since the agent was administered systemically. Previous observations in pylorus-ligated rats also indicated that the SSRIs, fluoxetine and sertraline, as well as the heterocyclic drug trazodone given systemically, exacerbate gastric injury in rats treated with indomethacin[27]. This contrasts with the gastric protective effects of tricyclic agents, which are likely to result from their anticholinergic and antihistaminic properties[28]. In man, the administration of SSRIs are likely to be associated with increased risk of gastrointestinal bleeding. This is especially the case in the elderly and in those on NSAID therapy[29]. In anesthetized rats, fluoxetine and sertraline increased gastric acid secretion[30]. This contrasts with the inhibition of gastric acid secretion by *H. perforatum* in the present study and suggest that other mechanisms mediate the gastric irritant action of *H. perforatum*.

In summary, the present study indicates that *H. perforatum* possesses anti-inflammatory and antinociceptive properties, thereby suggesting that the extract might be useful in the management of inflammatory pain. Similar to the SSRIs, the agent causes gastric irritation and might aggravate that of NSAIDs.
